# Evaluation of a New Real-Time Dosimeter Sensor for Interventional Radiology Staff

**DOI:** 10.3390/s23010512

**Published:** 2023-01-03

**Authors:** Kenshin Hattori, Yohei Inaba, Toshiki Kato, Masaki Fujisawa, Hikaru Yasuno, Ayumi Yamada, Yoshihiro Haga, Masatoshi Suzuki, Masayuki Zuguchi, Koichi Chida

**Affiliations:** 1 Course of Radiological Technology, Health Sciences, Graduate School of Medicine, Tohoku University, 2-1 Seiryo, Aoba-ku, Sendai 980-8575, Japan; 2 Department of Radiation Disaster Medicine, International Research Institute of Disaster Science, Tohoku University, 468-1 Aramaki Aza-Aoba, Aoba-ku, Sendai 980-0845, Japan; 3 Department of Radiology, Sendai Kousei Hospital, 4-5 Hirose-machi, Aoba-ku, Sendai 980-0873, Japan

**Keywords:** radiation protection and safety, fluoroscopy, interventional radiology (IVR), fluoroscopically guided interventional procedures, percutaneous coronary intervention (PCI), eye lens dose, occupational radiation exposure, X-ray examination, real-time radiation sensor

## Abstract

In 2011, the International Commission on Radiological Protection (ICRP) recommended a significant reduction in the lens-equivalent radiation dose limit, thus from an average of 150 to 20 mSv/year over 5 years. In recent years, the occupational dose has been rising with the increased sophistication of interventional radiology (IVR); management of IVR staff radiation doses has become more important, making real-time radiation monitoring of such staff desirable. Recently, the i3 real-time occupational exposure monitoring system (based on RaySafe^TM^) has replaced the conventional i2 system. Here, we compared the i2 and i3 systems in terms of sensitivity (batch uniformity), tube-voltage dependency, dose linearity, dose-rate dependency, and angle dependency. The sensitivity difference (batch uniformity) was approximately 5%, and the tube-voltage dependency was <±20% between 50 and 110 kV. Dose linearity was good (R^2^ = 1.00); a slight dose-rate dependency (~20%) was evident at very high dose rates (250 mGy/h). The i3 dosimeter showed better performance for the lower radiation detection limit compared with the i2 system. The horizontal and vertical angle dependencies of i3 were superior to those of i2. Thus, i3 sensitivity was higher over a wider angle range compared with i2, aiding the measurement of scattered radiation. Unlike the i2 sensor, the influence of backscattered radiation (i.e., radiation from an angle of 180°) was negligible. Therefore, the i3 system may be more appropriate in areas affected by backscatter. In the future, i3 will facilitate real-time dosimetry and dose management during IVR and other applications.

## 1. Introduction

Medical radiation (patient radiation doses and occupational exposure) is a major problem in radiation medicine [1,2,3,4,5,6,7,8,9,10].

Interventional radiology (IVR) plays a major role in disease diagnosis and treatment. IVR is performed using X-ray imaging equipment, catheters, and needles [11,12,13,14].

The procedural times of sophisticated IVR and other procedures have lengthened, increasing the radiation dose and making radiation control very important [15,16,17,18,19,20,21,22].

**Figure 1:**
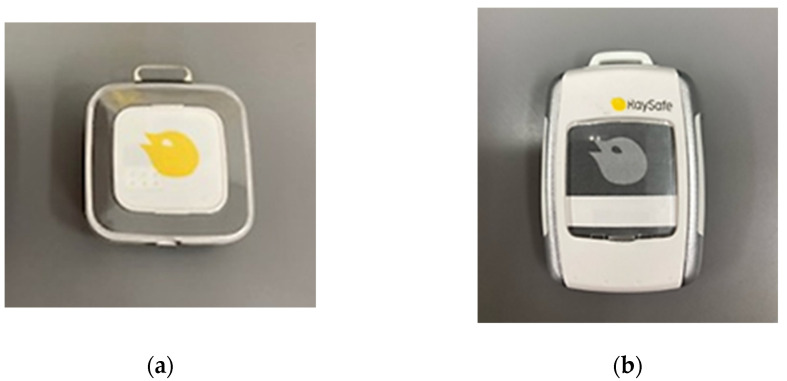
(**a**) The RaySafe i2 sensor (44 × 45 mm); (**b**) The RaySafe i3 sensor (40 × 58 mm). Recently, the i3 sensor has replaced the former i2 sensor.

The 2011 statement of the International Commission on Radiological Protection (ICRP) reduced the eye-lens exposure limit (the occupational dose) from 150 to 20 mSv/year [23]. It is expected that some medical staff will exceed this, and thus it is essential to evaluate the dose to the lens. Currently, in Japan, radiation doses delivered to medical staff during IVR and other procedures are assessed principally using radiation-monitoring badges attached to the neck, chest, or abdomen. Such badges measure cumulative doses over a long period (e.g., 1 month), not the dose associated with each examination or procedure. We think that it may be difficult to reduce the occupational dose further in the absence of real-time radiation monitoring in IVR. Originally, the RaySafe i2 (i2) real-time dosimeter was used (Figure 1a). This has been replaced by the RaySafe i3 (i3) (Figure 1b) [24,25]. According to the manufacturer, the i3 is better than the i2 in terms of scattered-radiation detection, easy battery replacement, and analytical performance. Here, we compared the i3 and i2 dosimeters (sensors) [26].

## 2. Materials and Methods

### 2.1. The i3 Dosimeter

**Figure 2:**
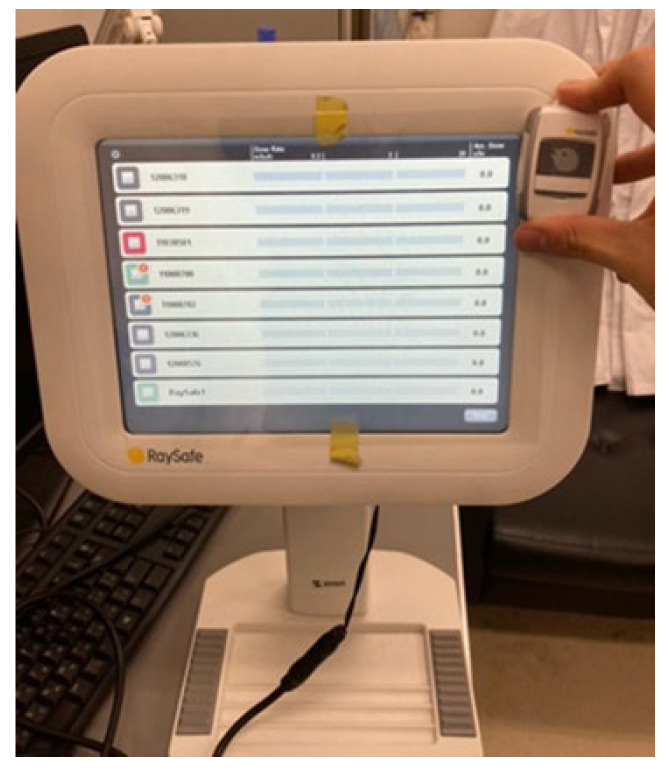
A schematic of the i3 system (left: base station with 10.4-inch display; right: i3 dosimeter sensor). The system comprises a wireless i3 sensor (solid-state semiconductor detector) that transmits the scattered-radiation dose to the base station. The i3 sensor is small (40 × 58 mm, 34 g) and thus easy to carry during interventions.

Like the i2 dosimeter, the i3 measures scattered radiation (1 cm dose equivalent) in real-time, displaying both the dose rate and the cumulative dose (Figure 2). The dose rate is displayed as a red, yellow, or green bar, from the highest to the lowest dose, that is refreshed at 1-s intervals; a glance is sufficient to determine whether the dose rate is high. The i3 system stores dose data; a chronological dose history can be viewed and subjected to time-series analysis using a PC running dedicated software. Additionally, the i3 dosimeter uses a replaceable battery as opposed to the i2 dosimeter model in which the battery is non-replaceable.

### 2.2. Fundamental Evaluation

**Figure 3:**
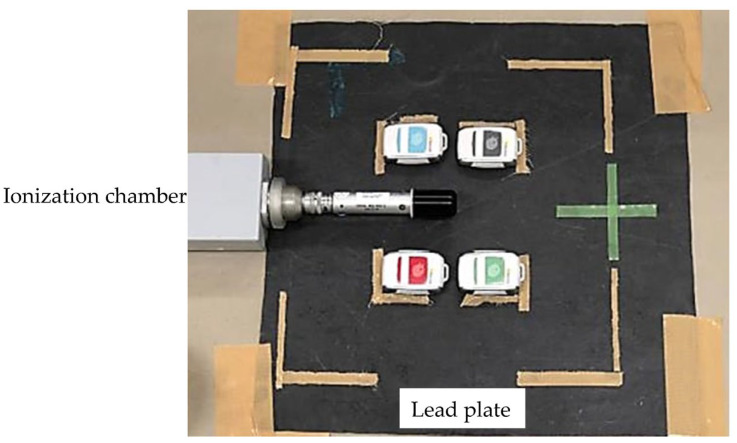
Experimental layout. The exposed area (radiation field) at the i3 entrance was 30 × 30 cm.

We used a diagnostic X-ray system featuring a high-frequency inverter generator (DHF-155H, Hitachi) to evaluate the i3 system. The total filtration of this X-ray system was 3.6-mm-aluminum equivalent. The distance from the X-ray tube to the i3 system was 180 cm, and the exposed area (the radiation-field size) at the i3 entrance was 30 × 30 cm. Variation in the sensitivity (batch uniformity) of i3 systems was evaluated by directly irradiating four i3 systems and an ionization chamber dosimeter simultaneously (Figure 3). Fluoroscopy was performed for 1 min under three conditions: (1) 65 kV tube voltage and 1 mA tube current, (2) 65 kV tube voltage and 0.1 mA tube current, and (3) 40 kV tube voltage and 0.1 mA tube current. These X-ray output conditions were to simulate scattered radiation (i.e., a low dose rate). To confirm reproducibility, all measurements were performed 10 times. An ionization chamber calibrated using the Japan national standard exposure dose (thimble type 6 mL; Model-9015, Radcal) was used to confirm the stability of the instrument. 

The tube-voltage dependency of the i3 system was evaluated under the same geometric conditions as employed when assessing batch uniformity. Fluoroscopy was continuous over 1 min, as shown in Table 1. Three measurements were made at each tube voltage, and the averages were calculated. The tube-voltage dependency for the i3 dosimeter was the ratio of the average i3 value to that of the ionization chamber dosimeter.

Dose linearity was measured using the integrated dose for X-ray irradiation of the four i3 systems under the same geometric conditions employed to evaluate batch uniformity. The fluoroscopy conditions were a 65 kV tube voltage, 1.6 mA tube current, and 15 min fluoroscopy time. The i3 integrated doses were recorded at 1, 2, 4, 6, 8, 10, 12, 14, and 15 min after fluoroscopy commencement. The experiment was repeated three times. For each dataset, the coefficient of determination (R^2^) was calculated by approximating linearity using the least-squares method in Microsoft Excel.

The dose-rate dependency of the i3 system was measured at 11 different dose rates ranging from 20–500 mGy/h. The dose-rate dependency was the ratio of the mean i3 value to that of the ionization chamber dosimeter. Copper plates were attached to the X-ray tube entrance when measuring low dose rates. The i2 dosimeter was evaluated under the same conditions, and the dose-rate dependencies of the i2 and i3 systems were compared.

The limit of radiation detection of the i2 and i3 systems was evaluated using fluoroscopy scatter radiation from an acrylic phantom. The fluoroscopy tube voltages were 60, 80, and 100 kV, and the fluoroscopy durations were 3, 10, and 60 s.

**Figure 4:**
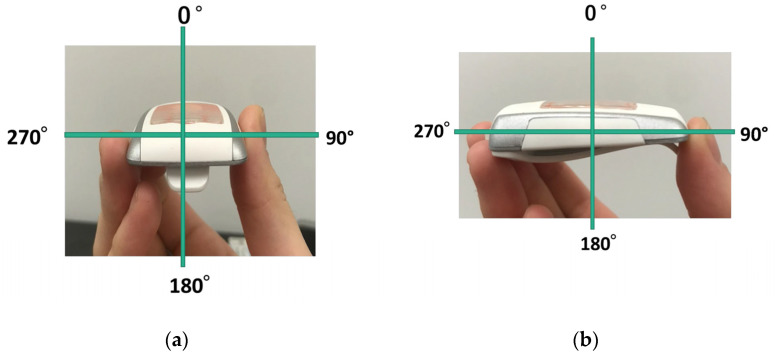
Experimental layout for evaluation of angle dependency: (**a**) Horizontal; (**b**) Vertical.

A digital cine single-plane X-ray system (Infinix Celeve-I: INFX-8000F, Toshiba Medical) was used to measure angle dependency. The i3 sensor was placed 75 cm from the focal point of the X-ray tube and irradiated in free air. The dependency of the i3 sensor on the X-ray beam angle in air was measured at 0, ±15, ±30, ±45, ±60, ±75, ±90, ±135, and 180° along the vertical and horizontal axes under identical X-ray conditions (70 kV, HVL, 2.7 mm aluminum, 10 mA, 5 ms); the 0° measurement served as the reference value. The experiment was repeated five times at each angle (Figure 4). This method is that of Inaba et al. [26].

## 3. Results

### 3.1. Fundamental Evaluation

Table 2 shows the variations in sensitivity (batch uniformity). For the first condition, the reproducibility of each detector (average coefficient of variation (CV), CV = standard deviation/mean measurements) was 2.099% (range 1.380–3.192%), and the batch uniformity (CV of each i3 sensor measurement) was 3.239%. For the second condition, the reproducibility of each detector was 2.216% (range 1.934–2.398%) and the batch uniformity was 3.431%. For the third condition, the reproducibility of each detector was 4.847% (range 2.291–6.913%) and the batch uniformity was 8.141%.

**Figure 5:**
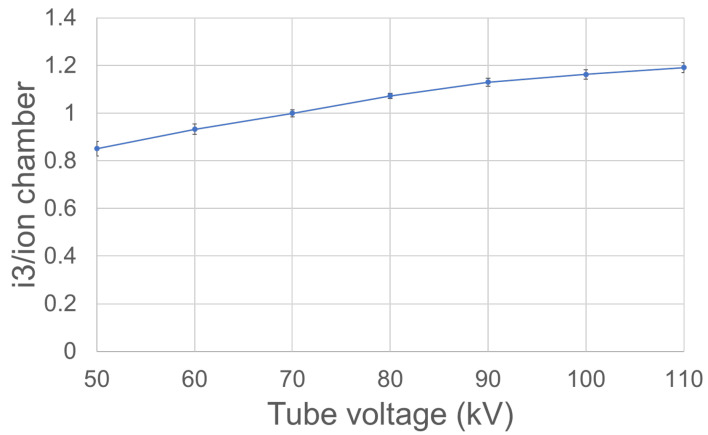
Tube-voltage dependencies of the i3 system (vertical axis: i3 measurements/ionization chamber measurements; horizontal axis: tube voltage).

Figure 5 shows the i3 tube-voltage dependency with respect to that of the ionization chamber dosimeter. Although the i3 value decreased with decreasing tube voltage, the difference in the i3 and chamber dosimeter values was <±20%, using the 70 kV measurement as the reference value.

**Figure 6:**
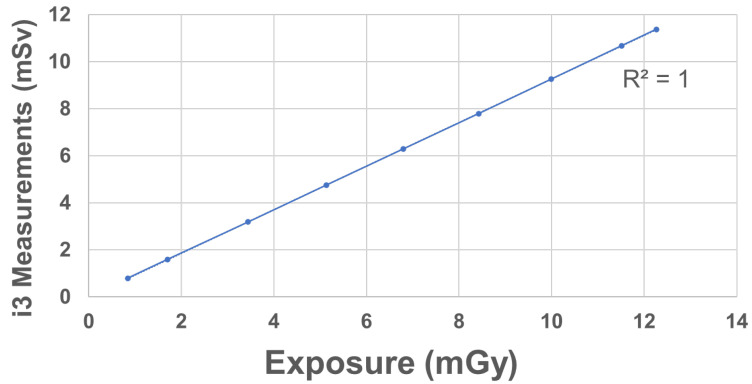
Dose linearities of the i3 system (vertical axis: i3 measurements; horizontal axis: ionization-chamber measurements).

Figure 6 shows the dose linearities. The R^2^ was 1.00.

**Figure 7:**
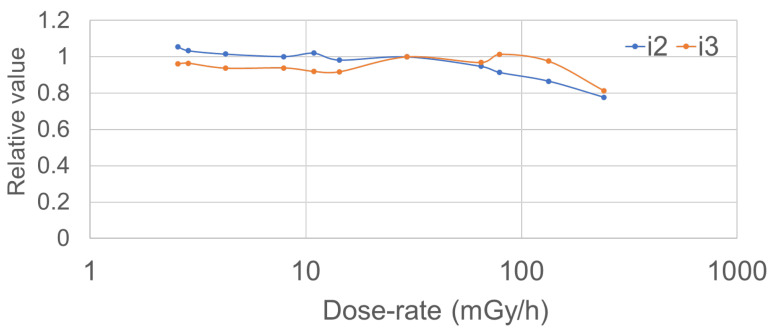
Dose-rate dependencies (vertical axis: i3 or i2 measurements/ionization-chamber measurements; horizontal axis: ionization-chamber measurements).

Figure 7 shows the dose-rate dependencies. The dose per hour is shown on the horizontal axis, and the ionization-chamber dosimeter reading divided by those of the i2 or i3 is shown on the vertical axis. At low dose rates, the i2 and i3 responses were similar. At very high dose rates (250 mGy/h), both the i2 and i3 evidenced dose-rate dependency (~20%).

Table 3 shows the limit of radiation detection of the i3 and i2 dosimeters. The i3 dosimeter showed better performance for the lower radiation detection limit compared with the i2 system.

### 3.2. Angle Dependency

**Figure 8:**
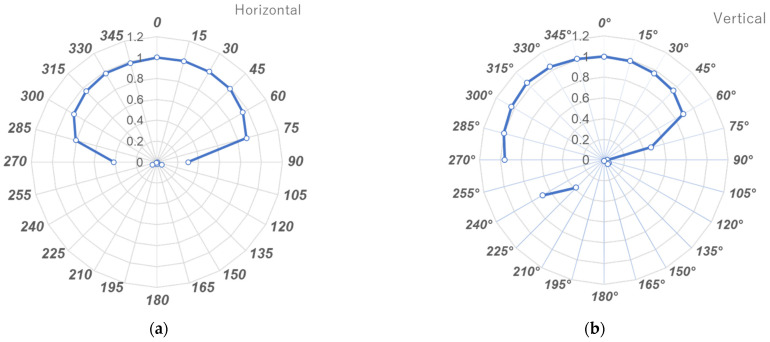
Angle dependency of the i3 sensor: (**a**) Horizontal plane; (**b**) Vertical plane.

**Figure 9:**
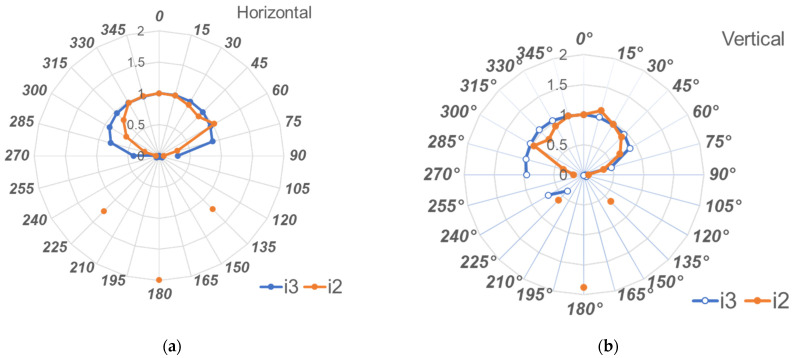
Angle dependencies of the i3 and i2 sensors: (**a**) Horizontal plane; (**b**) Vertical plane.

Figure 8a,b show the results in the horizontal and vertical directions, respectively. All doses are expressed as relative values, where 1 is the dose at 0°. In the horizontal direction, the i3 exhibited a reliable dose response from 0 to ±75° with a sensitivity >80%. In the vertical direction, the i3 exhibited a reliable dose response from 0 to +65° and 0 to +270° (−90°) with a sensitivity >80%. Figure 9a,b show the i2 sensitivities in the horizontal and vertical directions, respectively. i3 evidenced better angle dependency than that of i2. Furthermore, using the i2 sensor, the influence of backscattered radiation must be considered.

## 4. Discussion

Safety measures to prevent radiation exposure are important due to the risk of radiation-induced injuries, such as skin damage in patients and cataracts in medical staff [27,28,29,30,31,32,33,34,35,36,37]. Therefore, increasing attention is being paid to radiation safety and protection for patients and medical staff, particularly related to IVR [38,39,40,41,42,43,44,45,46,47].

Radiation monitoring badges (e.g., glass badges) and pocket dosimeters are used extensively to assess radiation doses to medical staff. Glass badges measure long-term exposure, but they cannot be used for real-time measurements. Pocket dosimeters measure doses in real-time, but must be constantly checked. Unlike the i3 system, pocket dosimeters do not display doses in real-time on a monitor. Dosimeters placed in the vicinity of the lens, such as the Eye-D and DOSIRIS, can also be used [26,48,49,50,51,52]; the passive DOSIRIS dosimeter, which does not provide real-time monitoring, was designed to measure the lens dose, but the real-time i3 system may be more effective for reducing occupational doses. The use of dosimeters such as the i3 will be valuable in situations such as IVR, in which exposure doses are high and instantaneous monitoring is required [53,54,55,56,57,58].

Real-time monitoring is important to minimize the exposure of medical staff and to ensure adequate protection [59,60,61,62,63,64]. To the best of our knowledge, this is the first detailed fundamental study of the ability of the i3 dosimeter to monitor the real-time occupational doses of IVR staff. The reproducibility of each i3 system and the batch uniformity among the systems were both approximately 5%, thus comparable with or better than those of the i2 system. 

In terms of tube-voltage dependency, the lower the tube voltage, the slightly lower the i3 value. However, if the ratio of the values measured at 70 kV was set to 1, the difference between the 50 and 110 kV values was <±20%, thus well within the ±25% range of the Raysafe instruction manual [25]. 

The dose linearity of the i3 system was good (R^2^ = 1). It was reported previously (Inaba [26]) that the i2 dose linearity is also good (R^2^ = 1). The i2 and i3 systems may be similar in this respect.

In terms of the dose-rate dependency, a decrease in sensitivity was observed at high dose rates (e.g., 250 mGy/h) for both dosimeters. As the scattered radiation received by an IVR physician is lower than this, we do not perceive a clinical problem. In detail, the diagnostic reference level (DRL) of the patient reference fluoroscopy dose rate during IVR in Japan (Japan DRL2020 [65]) is 17 mGy/min (i.e., 102 mGy/h). An IVR physician may receive between 1/1000 and 1/500 of the patient entrance dose, so, it has been thought that IVR physicians are not exposed to high dose rates (e.g., 250 mGy/h).

**Figure 10:**
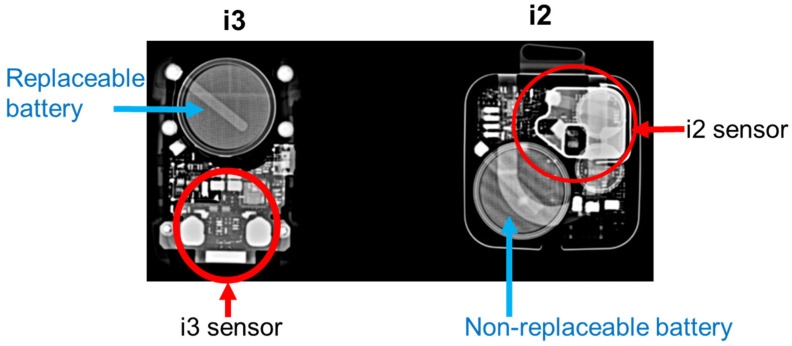
X-ray photographs for the i3 and i2 dosimeters.

The angle dependency of the i3 system was good in both the horizontal (0 to ±75°) and vertical (0° to +65° and –90°) axes. The angle dependency of the i3 was better than that of the i2. Regarding the semiconductor sensor and the internal structure of the dosimeter, there is no detailed information disclosure from the manufacturer. Figure 10 shows X-ray photographs from the i3 and i2 dosimeters. We speculate that the X-ray sensor of the i3 dosimeter has improved angle dependency by being placed at the bottom of the sensor compared to the i2 dosimeter in which the sensor is located at the upper right side.

Behind the sensor, i2 sensitivity almost doubled, whereas i3 was insensitive. When a dosimeter is mounted on the head or neck, backscattering must be considered. As the i3 is insensitive behind the sensor, such effects can be ignored.

The basic performance of the i3 system was thus equal to or better than that of the i2 system; the i3 should be preferred by medical staff. Although pocket dosimeters can measure doses in real-time, the i3 dosimeter is better because a glance at the display reveals the current dose, increasing radiation awareness.

In summary, the dose limits for medical personnel have been reduced in many countries; in Japan, the dose limit was significantly reduced from 150 to an average of 20 mSv/year over a 5-year period. Personal dose management is becoming increasingly important. Currently, badges (e.g., glass badges) and pocket dosimeters are used by medical staff; in the future, real-time dosimeters may become more important, especially in IVR. We previously reported the basic performance of the former i2 system (i2 sensor). Recently, a new i3 dosimeter (sensor) was developed to replace the i2 sensor. Here, we evaluated the basic performance of the new sensor (i3 system). The results show that the basic performance of the new i3 sensor is the same as or better than that of the i2 sensor. To date, it has not been possible to determine the chronological dose history (e.g., the dose rate, and exposure duration); the i3 system enables history determination at a glance. The i3 dosimeter is appropriate for clinical use, exhibiting especially good angle dependency. Such dosimeters will remain important in the future.

## 5. Conclusions

The i3 dosimeter performs as well as, or better than, the i2 dosimeter. The angle dependency of the i3 is particularly good. Furthermore, unlike the i2, the i3 can be used in areas exposed to backscatter. In the future, i3 will facilitate real-time dosimetry and dose management during IVR and other applications.

## Figures and Tables

**Table 1:** Fluoroscopic X-ray tube voltage and half value layer.

Tube Voltage (kV)	50	60	70	80	90	100	110
Half value layer (mmAl)	2.0	2.4	2.8	3.2	3.7	4.15	4.7

**Table 2:** Variations in sensitivity (reproducibility, batch uniformity).

	Reproducibility (%)	Batch Uniformity (%)
condition (1)	2.099 (range 1.380–3.192)	3.24
condition (2)	2.216 (range 1.934–2.398)	3.43
condition (3)	4.847 (range 2.291–6.913)	8.14

**Table 3:** Low radiation detection limit of the i2 and i3 systems.

Tube Voltage	60 kV			80 kV			100 kV		
Fluoroscopy Duration	3 s	10 s	60 s	3 s	10 s	60 s	3 s	10 s	60 s
i3 measurements (μSv/h)	57.5	30.5	19.5	59.6	34.5	21.3	48.4	27.3	16.6
i2 measurements (μSv/h)	185.7	46.3	41.3	101.3	31.2	35.2	99.8	39.8	22.2

## Data Availability

Not applicable.
